# Pharmacokinetic and pharmacodynamic modeling of anti-plasmodial drugs mefloquine plus artesunate: insights on translational application

**DOI:** 10.1128/aac.01717-25

**Published:** 2026-02-12

**Authors:** Sabiha R. Mim, Valdeene Vieira Santos, Laiz Campos Pereira, Kiana Pica, Aline Lorena Lourenço dos Santos Miranda, Helenita Costa Quadros, Diogo Rodrigo Magalhaes Moreira, Francine Johansson Azeredo

**Affiliations:** 1 Center for Pharmacometrics and Systems Pharmacology, Department of Pharmaceutics, College of Pharmacy, University of Florida 662907, Orlando, Florida, USA; 2 Pharmacy Graduate Program, College of Pharmacy, Federal University of Bahia 28111, Salvador, Brazil; 3 Fiocruz, Instituto Gonçalo Moniz 42509 , Salvador, Bahia, Brazil; 4 Department of Technology and Social Sciences, State University of Bahia, Juazeiro, Bahia, Brazil; Providence Portland Medical Center, Portland, Oregon, USA

**Keywords:** preclinical efficacy, translational pharmacology, PK/PD modeling, antimalarial combination therapy

## Abstract

A combination between artesunate (AS) and mefloquine (MQ) (ASMQ) is widely employed for the treatment of uncomplicated *P. falciparum*. Despite this, pharmacokinetic (PK) and underlying relationship between PK and pharmacodynamic (PD) are relatively less known than other standard combination therapy. This study aimed to develop population PK models for ASMQ combination therapy in *P. berghei*-infected mice and to further characterize the PK/PD relationships by assessing the impact of different dosing strategies through a model-based simulation. Plasma PK was assessed in infected mice receiving a single oral dose of 100 mg/kg AS and 55 mg/kg MQ after allometric scaling to mice dose. A two-compartment model with first-order absorption and linear elimination best described both drugs' concentration-time profiles. PK/PD modeling, performed using a turnover model in MonolixSuite 2024R1, revealed IC₅₀ values of 10.93 nM for artesunate and 29.1 nM for mefloquine, indicating strong potency. Simulations demonstrated that both ASMQ dosing regimens (100/55 and 25/55 mg/kg) resulted in comparable parasitemia suppression (~85%) and sustained efficacy. In contrast, AS monotherapy exhibited a rapid initial parasite clearance, but this was followed by a parasite recrudescence. These findings underscore the value of combination therapy and highlight the utility of integrated PK/PD modeling to inform antimalarial treatment optimization in preclinical studies and support translational application.

## INTRODUCTION

In 2023, there were an estimated 263 million cases of malaria, an increase of 11 million more cases than estimated in 2022 (1). Compared with other parasitic diseases, malaria is considered one of the most serious diseases with the highest mortality rate, being an important global health problem in tropical and subtropical regions (2).

For treatment of *Plasmodium* spp. infections, several commonly used antimalarials are available, such as chloroquine, amodiaquine, quinine, mefloquine, halofantrine, lumefantrine, primaquine, atovaquone, in addition to artemisinin-based drugs, dihydroartemisinin, artesunate, and artemether. For uncomplicated *P. falciparum*, artemisinin-based combination therapies (ACTs) are an association of one artemisinin derivative with a slower elimination antimalarial (2, 3). ACT, composed of artesunate and mefloquine, can be as safe and effective for the treatment of uncomplicated malaria caused by *P. falciparum* when compared with other ACTs, such as artemether plus lumefantrine (4). Unfortunately, malaria parasites are becoming resistant to antimalarial medications. *P. falciparum* already shows reduced *in vivo* susceptibility to artesunate and mefloquine monotherapy between Thailand and Cambodia, historically a site of emerging resistance to antimalarial drugs, being characterized by low parasite clearance *in vivo* (5, 6). Despite the administration of high-dose mefloquine, treatment failure rates approached 50%, prompting the discontinuation of mefloquine monotherapy and its replacement with artesunate (AS) and mefloquine (MQ) (ASMQ) combination therapy at a regimen of 4 mg/kg/day of artesunate and 25 mg/kg of mefloquine in Thai patients (7).

The World Health Organization recommends a standard fixed-ratio regimen of ASMQ of 100/200 mg of two tablets or a target dose of 4 mg/kg AS and 8.3 mg/kg MQ once daily for adults and children over 6 years of age (8). Although the ASMQ combination is widely used in Latin America (9), pharmacokinetic (PK) and any underlying relationship between PK and pharmacodynamic (PD) are relatively less known than other standard combination approaches. Moreover, an open-label, multicenter clinical trial of ASMQ combination therapy in children conducted by Drugs for Neglected Diseases initiative (DNDi) has revealed a considerable variability in drug exposure and suggested modified absorption rates in the population pharmacokinetic model (9).

Major problems with the ASMQ combination are the fact that MQ can present erratic absorption, adverse toxic effects, in addition to the potential for a shortage of MQ supply. Conceivably, adjusting the MQ dosing could overcome these liabilities.

In light of recent investigations studying fractional curative killing, PK and PK/PD relationships of combination therapies for uncomplicated malaria (10–13), here we sought to study the PK and PK/PD relationships for a new fixed ratio of ASMQ at 2:1 (100 mg/kg artesunate and 55 mg/kg mefloquine). We hypothesized that by reducing the dosing of MQ while maintaining the AS dosing, it could be as effective as the standard fixed ratio of ASMQ at 1:2. Importantly, this new proposed fixed ratio could overcome many liabilities of MQ and of potential translation in Latin America, a setting where partial artemisinin-resistance phenotype is not prevalent.

Here, the preclinical study of oral ASMQ was performed in *P. berghei*-infected mice to determine the drug plasma PK profile of this combination therapy. To establish a robust PK/PD relationship, single drug dosing efficacy in mice was performed in different fixed ratios, including the standard fixed ratio of ASMQ at 1:2 as well as temporal analysis in parasitemia reduction.

Finally, a robust mathematical modeling was assessed. Mathematical models can be developed to obtain information about the transmission of diseases and what influences their prevention and treatment. Among them, PK/PD models in preclinical studies enable the use of *in vitro* drug efficacy data, parasite growth dynamics, and animal studies to extrapolate drug exposure and efficacy for studies in humans, in addition to allowing the evaluation of synergy in drug combinations (14). PK/PD modeling has been successfully applied to describe the efficacy and predict effective antimalarial regimens (15), with emphasis on the model describing MQ for the treatment of patients with malaria from sensitive and resistant *P. falciparum* strains (16) and simulation of PK and PD curves based on chosen values of PK-PD parameters from clinical studies in the literature (17).

Overall, our present study aimed to develop a PK/PD model of the ASMQ combination therapy administered together against *P. berghei* in a preclinical model to capture the antiparasitic effect after drug administration and serve as a rational tool that can provide information on ways to optimize malaria treatment in humans (18).

## RESULTS

### Characterization of ASMQ efficacy in mice

**Fig 1:**
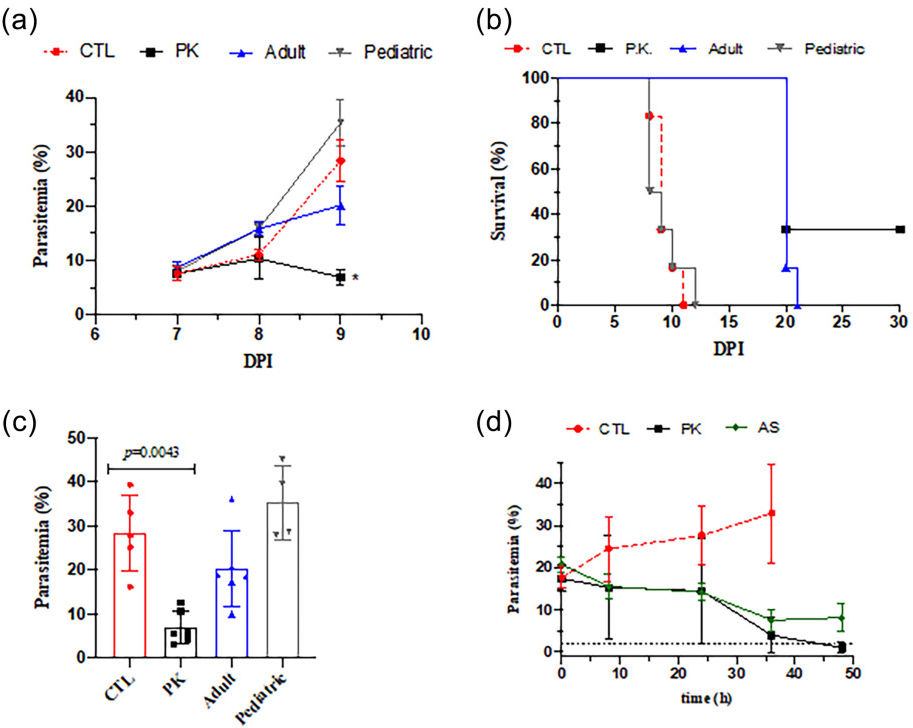
Efficacy of ASMQ in mice. **a and b** show the parasitemia and animal survival in *P. berghei*-infected Swiss mice. Drug treatment was given orally by a single dose, parasitemia was determined by flow cytometry and animal survival was monitored daily. **c** shows parasitemia from **a** determined at 7–9 days (48 h) post-infection. **a-c **are experiment #1 (*n* = 6/group). **d** displays the parasitemia measured at indicated post-treatment (experiment #2; (*n* = 6/group). CTL, untreated infected mice; DPI = days post-infection; FR = Fixed dose of 200 mg/kg MQ plus 100 mg/kg AS; AS (FR) = monotherapy using matched dose of FR of 100 mg/kg AS. Statistical analysis was conducted using the Mann-Whitney rank test. Unless indicated, ^##^ or *P < 0.05. ^#^*P* < 0.05 (log-rank, Mantel-Cox test).

Intraperitoneal inoculation of ANKA luc/gfp strain of *P. berghei* in Swiss mice led to increased parasitemia and a decreased animal survival (Fig. 1a). A single oral dose by gavage given at 6 days post-infection (DPI) of the new fixed ratio of ASMQ at 2:1 (100 mg/kg artesunate and 55 mg/kg mefloquine), also referred to as therapeutic PK regime, was statistically significant to reduce parasitemia and to increase animal survival in comparison to untreated control (Fig. 1a and b). This PK regime cured 40% mice. Subsequent allometric dosing of human adult and pediatric ASMQ in mice was calculated, and the proposed doses were tested in *P. berghei*-infected mice (Fig. 1a and b). Adult regimen (41 mg/kg artesunate and 20.5 mg/kg mefloquine) reduces parasitemia and increases animal survival at statistically significant levels; however, this adult regimen did not provide a cure. Finally, it was observed that pediatric ASMQ (2.5 mg/kg artesunate and 5.0 mg/kg mefloquine) did not reduce parasitemia (Tables S1 and S2).

Having ascertained that the therapeutic PK regime of ASMQ presents a significant efficacy, the underlying efficacy of this combination was investigated. MQ is categorized as a drug of moderate speed of activity. This drug is efficient in killing alltrophozoites stages of *P. falciparum,* and therefore, it has a speed of activity, which is slower than dihydroartemisinin but faster than atovaquone (19). Based on this, parasitemia was determined within different intervals after therapeutic PK regime of ASMQ and a matched dose of AS monotherapy (100 mg/kg AS) in order to ascertain the contribution of each drug in the speed in antiplasmodial activity. In comparison to the control group, the PK regime of ASMQ led to a decrease in parasitemia at 36 h post-treatment. A similar decrease in parasitemia at 36 h post-treatment was observed for the AS monotherapy. However, at 48 h post-treatment, parasitemia at the PK regime was within the limit of detection, while at 48 h post-treatment, AS monotherapy presented parasitemia higher than the limit of detection. Of note, parasitemia in AS monotherapy was notstatistically different between 36 h versus 48 h, denoting a recrudescence in parasite growth (Fig. 1c and d).

In another set of experiments, the ASMQ dosing regimen was scaled from the human therapeutic dose to a fixed ratio of 100 mg/kg AS and 200 mg/kg MQ. This standard regimen was evaluated for its temporal efficacy, and compared with the control group, it produced a marked reduction in parasitemia at 36 h post-treatment. At both times of 36 h and 48 h post-treatment, parasitemia was within the limit of detection. In contrast, a matched dose of AS monotherapy (100 mg/kg artesunate) run in parallel led to a decrease in parasitemia at 36 h but presented parasite re-growth at 48 h (Table S1).

It was further observed that the PK regime of ASMQ is more efficacious in reducing infection than a matched dose of AS monotherapy. Moreover, the PK regime of ASMQ is of similar efficacy to the standard fixed ratio regime of ASMQ. The difference between the therapeutic PK regime versus standard fixed ratio regime is that the PK regime reduces parasitemia to the levels near the limit of detection at 48 h, while the standard fixed ratio regime reduces this as early as 36 h. Moreover, examination of the temporal efficacy in parasitemia dropping indicates that the MQ component of ASMQ has little contribution to the initial dropping in parasitemia within 24 h, a time frame where *P. berghei* growth and re-invasion occurs. Based on this, MQ contribution relies on inhibiting remaining parasites once AS is eliminated in plasma. Second, the observed efficacy in a single dose of this newly proposed PK regime of ASMQ justifies the determination of its PK profile.

### Pharmacokinetic model

Following oral administration, the plasma concentration-time profiles of AS and MQ were best described by a two-compartment model with first-order absorption and linear elimination, providing a robust characterization of the observed data. In comparison, one- and three-compartment models did not adequately fit the PK data, based on visual inspection and diagnostic goodness-of-fit criteria. Residual unexplained variability was accounted for using a proportional error model for PK parameters and an additive error model for PD parameters.

Due to the sparse sampling design, variability between animals could not be distinguished from residual variability; therefore, inter-individual variability (IIV) was fixed to a minimal value of 0.001 to stabilize the model. As a result, formal covariate analysis was not performed, recognizing that IIV and residual unexplained variability cannot be reliably separated in this data set. All PK parameters for both artesunate and mefloquine were estimated with acceptable precision and low residual error (Tables 1 and 2). Furthermore, exposure parameters for mefloquine and artesunate in the combination therapy were comparable to those observed with monotherapy, supporting the consistency and plausibility of the model estimates (20–23).

**TABLE 1:** Estimated population pharmacokinetic parameters of mefloquine (MQ) following oral single administration of therapeutic PK therapy of ASMQ in *P. berghei*-infected mice

Parameter	Unit	Estimate	Relative SE (%)	95% CI
Absorption rate constant, Ka	1/h	0.28	24.4	0.18–0.45
Total clearance, CL	ml/h/kg	286.01	5.22	258.25–316.75
Volume of central compartment, V1	ml/kg	4039.63	11.2	3,254.16–5,014.7
Volume of central compartment, V2	ml/kg	41817.36	25.7	25,825.85–67,710.89
Inter-compartmental clearance, Q	ml/h/kg	20636.48	36.0	10,779.29–39,507.64
Proportional error model	NA^*a*^	0.71	18.5	0.50–1.01

^*a*^ NA, not applicable.

**TABLE 2:** Estimated population pharmacokinetic parameters of artesunate (AS) following oral single administration of therapeutic PK therapy of ASMQ in *P. berghei*-infected mice

Parameter	Unit	Estimate	Relative SE (%)	Bootstrap 95% CI
Absorption rate constant, Ka	1/h	0.6 (fixed)	NA^*a*^	NA
Total clearance, CL	ml/h/kg	3,285.52	11.9	2,607.25–4,139.84
Volume of central compartment, V1	ml/kg	1,921.4	14.0	1,467.33– 2,515.99
Volume of central compartment, V2	ml/kg	11,342.3 (fixed)	NA	NA
Inter-compartmental clearance, Q	ml/h/kg	19,210.83	6.39	16,954.46– 21,767.48
Proportional error model	NA	0.52	15.8	0.38–0.84

^*a*^ NA, not applicable.

**Fig 2:**
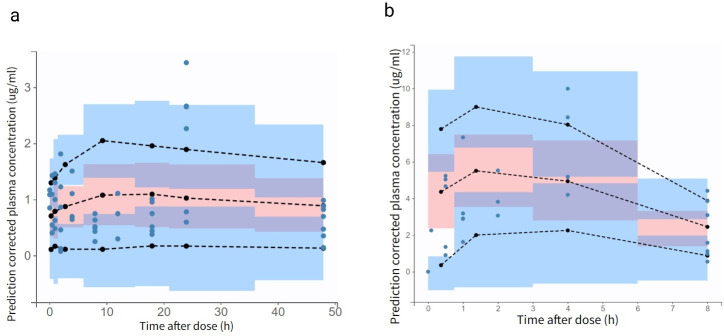
Prediction-corrected visual predictive checks (pcVPCs) for the final pharmacokinetic models of (**a**) mefloquine and (**b**) artesunate. Closed circles represent the prediction-corrected observed plasma concentrations. The black dashed lines indicate the median and 90% prediction interval (PI) of the observed data, overlaid on the model-simulated prediction intervals.

Goodness-of-fit plots for the final models of both AS and MQ demonstrated acceptable agreement between individual predictions and observed concentrations, with points closely aligned around the line of identity. The prediction-corrected visual predictive checks further indicated that most observed data fell within the model’s prediction intervals, supporting the adequacy of the model fit (Fig. 2). Additionally, the concentration-time profiles confirmed that the model appropriately captured the absorption and distribution phases, with only minor deviations, which are expected given the limited sampling points and inherent biological variability in the preclinical setting.

### Pharmacodynamic model

The pharmacodynamic response was described using an indirect response (turnover) model without a sigmoidal component, where drug exposure inhibits the production of the response variable (parasite count). The initial parasite inoculum at time zero was fixed at 17.8%, and the degradation rate constant was fixed at 0.08 h⁻¹, based on values reported by Burgert *et* al. (24) The PK/PD model for single-dose ASMQ combination therapy adequately captured the preclinical observations. In this mouse study, artesunate exhibited a relatively longer half-life, enabling the model to reflect its gametocidal activity, with an estimated IC₅₀ of 10.93 nM and a low residual standard error. In contrast, MQ demonstrated prolonged persistence within erythrocytes, with an estimated IC₅₀ greater than 30 nM, indicating sustained potency. For MQ, parasite sensitivity was reported to be approximately 1.5-fold above the cutoff level (IC₅₀ < 36  nM) (16), while AS IC₅₀ values have been shown to range from 0.9 to 60 nM (25). The developed PK/PD model, which estimated IC₅₀ values of 30 nM for mefloquine and 11 nM for artesunate (Table 3), is consistent with these reported ranges and supports the model’s ability to predict the expected clinical efficacy of both drugs.

**TABLE 3:** Estimated population PK-PD parameters from ASMQ combination therapy in *P. berghei*-infected mice

Parameters	Unit	Estimate	Relative SE (%)	Bootstrap 95% CI
Baseline response, R0	%	17.8 (fixed)	NA^*a*^	NA
Degradation rate, Kout	1/h	0.08 (fixed)	NA	NA
Half-maximal inhibitory concentration, IC_50_ (MQ)	ug/mL	0.011	11.2	0.0086–0.013
	nM	29.1	NA	25.6–31.7
Half-maximal inhibitory concentration, IC_50_ (AS)	ug/mL	0.0044	4.62	0.0040–0.0048
	nM	10.93	NA	8.32–14.31
Constant error model	NA	0.52	NA	NA

^*a*^ NA, not applicable.

### Impact of mefloquine and artesunate in combination treatment

**Fig 3:**
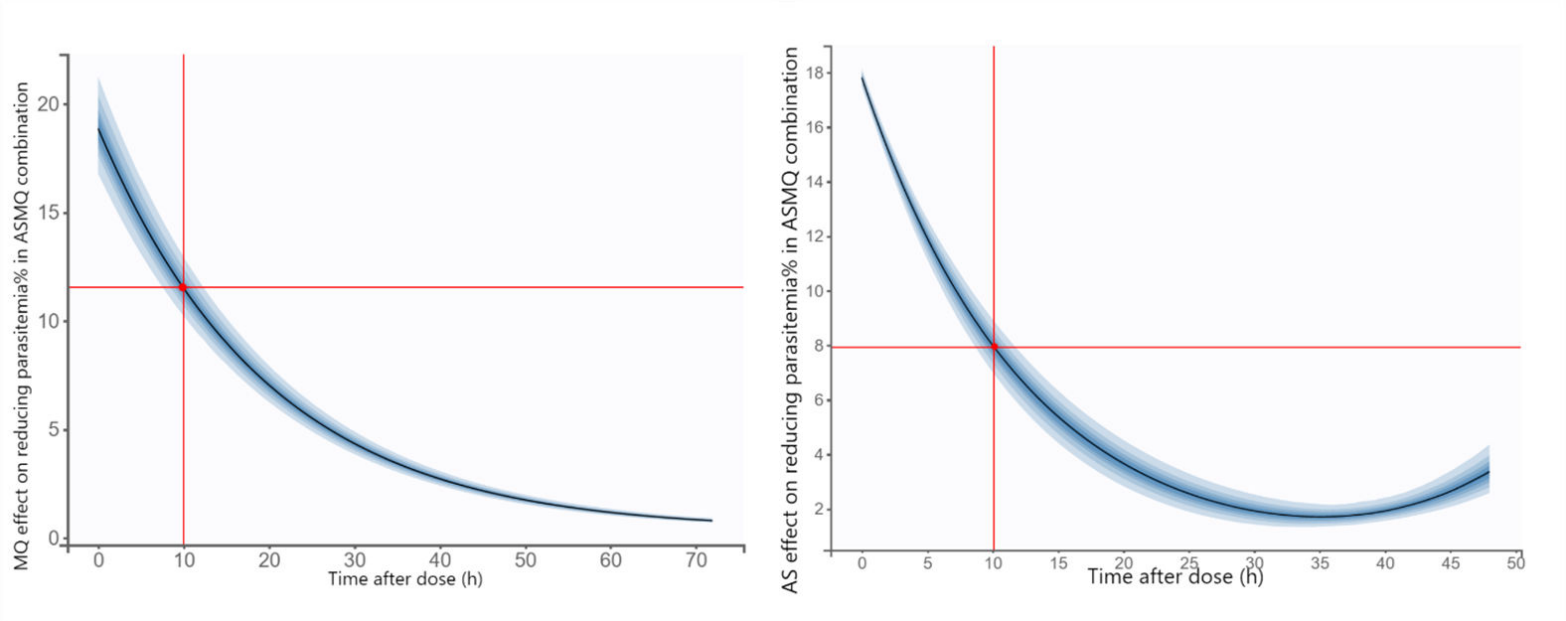
Simulated effect of mefloquine (left panel) and artesunate (right panel) on parasitemia reduction following a single dose of ASMQ combination therapy in mice. The black line represents the median predicted response, and the shaded area indicates the 5th to 95th percentile prediction interval.

Figure 3 presents the simulated impact of mefloquine (left panel) and artesunate (right panel) on parasitemia reduction following single-dose ASMQ combination therapy in mice. At 10 h post-administration, mefloquine lowered parasitemia from baseline to approximately 12%, while artesunate reduced it to about 8%. Due to its slower clearance, mefloquine maintained parasitemia suppression for >72 h after dosing. In contrast, artesunate produced a rapid but short-lived parasiticidal effect, with parasitemia levels showing a tendency to rebound as drug concentrations declined.

### Simulations to explore efficacy

**Fig 4:**
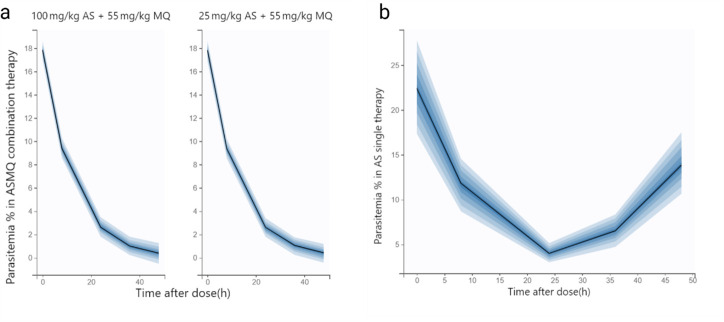
Simulated parasitemia percentage profiles generated using “true” pharmacokinetic parameters of mefloquine + artesunate (**a**) and artesuane alone (**b**). A total of 1,000 simulations were performed; the black line represents the median prediction, and the shaded area indicates the 5th to 95th percentile prediction interval.

Simulations comparing two different ASMQ dosing regimens (100/55 and 25/55 mg/kg of artesunate/mefloquine) revealed no significant differences in the predicted efficacy profile (Fig. 4a). These findings suggest that increasing the artesunate dose within this range does not lead to a meaningful improvement in treatment outcome, likely due to the high potency of both artesunate and mefloquine. Both dosing regimens resulted in a marked reduction, approximately 85%, in parasitemia levels during the first half of the *Plasmodium* spp. life cycle and continued to suppress parasitemia throughout the full cycle. This indicates that even the lower AS dose, when combined with MQ, is sufficient to maintain therapeutic efficacy. Additionally, simulations of AS monotherapy (100 mg/kg) demonstrated a less sustained response, with parasitemia declining by 78% at 24 h post-dose but rebounding to approximately 50% of baseline levels within 48 h (Fig. 4b). This regrowth pattern highlights the limitations of artesunate when used alone and emphasizes the added benefit of combination therapy in maintaining prolonged parasite suppression. The additive effect of combining artesunate’s rapid parasiticidal action with mefloquine’s longer-lasting effect supports the rationale for ASMQ use, particularly in reducing the likelihood of recrudescence and improving overall treatment durability.

## DISCUSSION

In comparison to Coartem (artemether plus lumefantrine), the PK/PD relationship of ASMQ was so far less understood. ASMQ is still the primary, and in many countries in Latin America, the sole treatment available for uncomplicated *P. falciparum*. In addition, a major potential problem is the shortage of MQ supply. Adjustment in MQ dosing is an important assessment.

The present analysis describes the pharmacokinetics and pharmacodynamic effects of ASMQ administered at a weight-based dose in a mouse model, with the objective of quantifying drug exposure and evaluating therapeutic efficacy. Although, in a preclinical study with a single-subject data set, noncompartmental or compartmental naïve pooled data (NPD) analysis is mathematically sufficient; however, a mixed-effects modeling (MEM) framework was employed to maintain consistency with standard pharmacometrics workflows and to enable mechanistic PK–PD linkage. Unlike pooled approaches, population PK modeling explicitly accounts for the kinetics of drug absorption, which is particularly relevant for orally administered therapies. Moreover, MEM has been shown to yield less biased parameter estimates than pooled or noncompartmental methods for sparse or single-sample-per-subject designs while preserving a model structure that can be readily extended to future population-level analyses.

Within this framework, the pharmacokinetics of MQ in mice were characterized by a slow clearance (286 mL/h/kg) and a moderate volume of distribution (4,039.63 mL/kg), indicating a relatively long half-life in infected mice. The estimated absorption rate constant (0.28 h⁻¹) was consistent with values observed in malaria human patients but slower than those reported in healthy volunteers. Since MQ plasma protein binding is comparable between rodents and humans, drug exposure can be reasonably extrapolated across species (20). Although simple allometric scaling, which provides functional approximation of human PK and may not fully capture MQ’s extended half-life due to the higher metabolic capacity of cytochrome P450 enzymes in mice compared to humans (26), it nevertheless predicted a half-life of approximately 90 h, aligning with values reported in some studies of patients with uncomplicated *P. falciparum* infection (27).

For AS, peak plasma concentrations observed in mice following oral ASMQ administration were substantially higher. The observed *C*max for AS in mice was approximately 6,500 ng/mL, which is nearly 10 times higher than the *C*max reported in rats receiving an equivalent single-agent dose. Interestingly, artesunate exposure in humans is comparatively lower, with *C*max values roughly sixfold lower than those observed in rats. Notably, Davis et al. (28) demonstrated that MQ co-administration significantly increased artesunate peak concentration (by approximately fourfold) in healthy male subjects, providing external clinical evidence supporting the plausibility of the elevated artesunate exposure observed experimentally in our animal model when administered as part of the ASMQ combination. From the compartmental modeling analysis of artesunate, this study also detected an effect of mefloquine on artesunate pharmacokinetic parameters, consistent with findings by Ferreira et al. (27), who reported that MQ’s volume of distribution and clearance are altered when co-administered with AS (9). Allometric scaling yielded an estimated human elimination half-life of approximately 2.6 h, reflecting an approximation of human disposition that accounts for known interspecies differences in metabolic capacity. In addition, formulation-related factors may influence the apparent prolongation of drug disposition (29).

Combining the pharmacokinetic parameters of both drugs allowed the pharmacodynamic effects on parasite reduction to be more accurately described using a turnover model rather than a direct response model. Although the final model represents a simplified version of the complex PK/PD interactions underlying ASMQ combination therapy in mice, it provides a useful framework for investigating the dose–exposure–parasite clearance relationship. Despite the study’s small sample size and the complexity of the biological system, this empirical modeling approach effectively captured the concentration–effect relationship during the blood stage. Integrating this mechanistic model with physiological understanding through a population PK/PD framework could further support the optimized use of oral ASMQ combination therapy and help address emerging drug resistance. Supporting this, studies by Kay et al. (30) and Nosten et al. (31) have shown that adding AS to MQ monotherapy can reduce treatment failure rates by approximately 95%.

In the final model, the estimated IC₅₀ for MQ was 29.1 nM, indicating sufficient potency to sustain parasite suppression during the intra-erythrocytic stage (asexual blood stages) for an extended period. In comparison, AS exhibited an estimated IC₅₀ of 10.93 nM, consistent with its higher intrinsic potency and rapid parasiticidal activity. Agomo et al. (32) also reported that the combination of AS and MQ provides effective gametocidal activity (sexual blood stages). Evaluation of the exposure–response profiles showed that the ASMQ combination substantially reduced the likelihood of recrudescence compared to artesunate monotherapy, which is more prone to treatment failure due to its rapid systemic clearance. This difference underscores the superiority of the ASMQ combination over monotherapy, supporting its role as a well-established and well-tolerated treatment option.

In our preclinical study, a 2:1 dose ratio of AS to MQ was used instead of the typical 1:2 ratio, primarily due to the higher cost and side effects (e.g., vomiting, neurological symptoms) associated with MQ. Despite this adjustment, the combination produced pharmacodynamic effects comparable to those observed with the conventional ASMQ dosing regimen and was well tolerated by the mice. Furthermore, dosing simulations demonstrated that a lower-dose regimen of 25 mg/kg AS combined with 55 mg/kg MQ yielded a similar therapeutic response in the earlier phase of infection. This suggests that, given the potency of both drugs and the nature of their effect, the treatment outcome is not strictly concentration-dependent within the tested range (33). However, AS monotherapy showed the pattern of recrudescence of parasitemia growth, which underscores the limitation of this monotherapy in sustaining parasite suppression and highlights the therapeutic advantage of ASMQ combination therapy. The addition of MQ, with its longer half-life and prolonged activity, complements the rapid but short-lived effect of AS, thereby improving overall efficacy and reducing the risk of treatment failure.

Overall, this preclinical mouse study enabled characterization of drug exposure and therapeutic efficacy for ASMQ combination therapy. Several analytical and modeling limitations merit consideration. Artesunate quantification was challenging due to its rapid hydrolysis and limited stability in aqueous and biological matrices, resulting in conversion to its active metabolite, dihydroartemisinin (DHA). Nonetheless, the derivatization-based analytical method employed allowed selective quantification of artesunate in plasma. From a modeling perspective, the sparse, destructive sampling design precluded reliable estimation of inter-individual variability; therefore, fixing variability to a small value was a pragmatic choice to ensure model stability. This represents an inherent limitation of the data set and highlights the need for confirmation in future studies employing richer sampling designs.

### Conclusions

This study highlights the value of integrated pharmacokinetic–pharmacodynamic analysis in elucidating the persistence and efficacy of artesunate–mefloquine combination therapy across the parasite life cycle. This approach can inform the development of other antimalarial combinations in preclinical settings and supports the evaluation of potential pharmacokinetic interactions and model-based simulations to predict treatment efficacy. Finally, to assess the impact of new ASMQ dosing regimens in mice, the PK and PD parameters were carefully incorporated while accounting for asexual blood stage parasitemia dynamics, treatment accessibility, and potential implications for health system costs.

## MATERIALS AND METHODS

### Mice and parasites

Male and female Swiss mice (6–8 weeks old; 18–22 g) were obtained from the Animal Resources Unit of the Instituto Gonçalo Moniz (Fiocruz, Salvador, Brazil) and housed in groups of four per cage under controlled environmental conditions, including a 12-h light/dark cycle, with free access to food and water. *P. berghei-*infected erythrocytes (strain ANKA gfp/luc) were thawed from frozen stocks and injected into Swiss donor mice.

### *P. berghei*-infected mice

Mice were inoculated intraperitoneally with 1 × 10⁶ parasitized erythrocytes suspended in 200 μL of 0.9% (w/v) saline solution and randomly divided into groups of four per cage. Parasitemia was monitored every 2 days by flow cytometry (BD LSRFortessa, BD Biosciences, USA) using SYTO-61 staining (Invitrogen, USA) and GFP signal. The relative reduction of parasitemia was calculated as: [(mean vehicle group) – (mean treated group)/(mean vehicle group)] × 100%). To ensure a humane endpoint, mice displaying symptoms of severe anemia were immediately euthanized. At the study endpoints, mice were euthanized by following induction of deep anesthesia using a ketamine/xylazine cocktail.

### Drug treatments

Mefloquine hydrochloride (MQ) was purchased from Sigma-Aldrich, and Artesunate was purchased from TCI Chemicals. Artesunate was dissolved in DMSO and then diluted in a dispersing solution (Kolliphor, 2%, polysorbate 80 2.5%, D-Sorbitol 2.5%, glucose 5%, and tween 20 2.5%; volume completed in phosphate buffer solution). The final proportion of DMSO was 5%. Mefloquine was directly diluted in the dispersing solution. Equal volumes of each drug were immediately mixed prior to drug administration in mice. A single administration by gavage inoculation (*P. Os*.) of 200 µL volume was given. Dosing was adjusted for individual animal weight.

### Determination of efficacy

*P. berghei*-infected mice at 6 days post-infection were divided into *n* = 5 at indicated groups (table below) and received drug administration by gavage inoculation (*P. Os*.) of 200-µL volume. The following parameters were evaluated: parasitemia at 7-, 8-, and 9-days post-infection, and 30-day post-infection (DPI) survival. Each experiment was run using no more than four groups per trial (Table S2). Each trial included an untreated infected control group.

### Pharmacokinetic experiments

The PK assessment was carried out in another experimental group of *P. berghei*-infected (*n* = 4 per datapoint). When parasitemia was equal to and/or greater than 5%, mice were divided into different groups and received a dose of the therapeutic PK regime, ASMQ combination at 100 and 55 mg/kg, respectively. At times of 0.083, 0.25, 0.5, 1, 1.5, 2, 4, 8, 12, 24, and 48 h, mice were euthanized, blood samples were quickly collected by puncture of the brachial plexus region, and placed in tubes containing heparin. After centrifugation, plasma was separated and stored at –80°C until analysis by high-performance liquid chromatography with diode array detector (HPLC-DAD).

### Bioanalytic methodology

For the quantification of artesunate and mefloquine in plasma samples, bioanalytical methodologies were validated in accordance with the U.S. Food and Drug Administration (FDA) Bioanalytical Method Validation Guidance for Industry (2018), including assessments of linearity, selectivity, precision, accuracy, and recovery assessment (34).

Quantification took place using an Agilent 1260 Infinity I HPLC system composed of a G1312B binary pump, G1367E autosampler, G1330B ALS thermostat, G1316A thermostatted column temperature (TCC), G4225A HiP degasser, and a G7115A wide-range diode array detector (DAD WR), all operated through Agilent OpenLab software. Chromatographic separation was achieved using a SUPELCOSIL LC-ABZ C18 reversed-phase column (150 × 4.6 mm, 5 µm, Sigma-Aldrich, USA).

The mefloquine quantification method was based on the work of Nogueira et al. (35). The mobile phase was a 50:50 (v/v) mixture of 25% methanol, 25% acetonitrile (ACN), 50% sodium phosphate buffer (pH 6.0 and 25 mM) with 0.5% triethylamine, delivered at 1 mL/min. Detection was performed at 220 nm, using a 15-µL injection volume and a column temperature of 35°C. Retention time for mefloquine was ~14.5 min. Calibration standards were made by spiking 10 µL of mefloquine solution into 90 µL of blank plasma. Mefloquine was extracted using ice-cold ACN at a 1:1 (v/v) ratio, following the method described by Izes et al. (36). The method was linear over the range of 0.75–24 µg/mL, with QC levels at 0.9, 4, and 20 µg/mL, and precision and accuracy within FDA limits.

Due to the lack of chromophores, artesunate quantification was performed using a derivatization method from Diawara et al. (37) to form the UV-detectable compound Q260. Despite its rapid hydrolysis to dihydroartemisinin (DHA) and limited stability, artesunate was selectively quantified using this derivatization-based analytical approach. After sample extraction with ACN in a 1:2 (v/v) ratio, based on Teja-Isavadharm et al. (38), the sample supernatant was evaporated and reconstituted in 100 µL of ACN, mixed with 400 µL of 0.2% (w/v) NaOH, incubated at 50°C for 30 min, cooled for 10 min, and treated with 100 µL ethanol and 0.2% acetic acid. Chromatography was performed using the same HPLC system and column. The mobile phase, optimized from Diawara et al. (37), consisted of 65% methanol and 35% 5 mM potassium phosphate buffer (pH 6.3), delivered at a flow rate of 1.0 mL/min. Detection was at 260 nm; retention time was approximately 9 min. AS solutions were prepared in ACN. Calibration and QC samples were prepared by spiking 10 µL of AS working solution into 90 µL of blank plasma, followed by the same extraction and derivatization procedures applied to study samples. Linearity was observed over the range of 2.5–40 µg/mL, with QC samples at 3.75, 7.5, 12.5, and 30 µg/mL, all within the FDA-accepted limits.

### Population PK-PD model development

Population pharmacokinetic analyses for artesunate and mefloquine were conducted independently to account for their distinct pharmacokinetic profiles. Subsequently, two separate PK/PD analyses were carried out. The primary analysis focused on characterizing the PK/PD relationship of artesunate as a monotherapy. This was followed by a secondary analysis incorporating data from the ASMQ combination therapy to evaluate potential interaction effects and dynamics of the combination. In the development of the ASMQ combination PK/PD model, individual pharmacokinetic parameters for mefloquine were derived from a previously established PK model and incorporated as fixed inputs. Parameter estimation was performed with residual unexplained variability modeled as a proportional error. Due to the single-subject nature of the data set, inter-individual variability could not be estimated and was therefore fixed to a small value under the assumption of a log-normal distribution. This strategy was employed to enhance model stability while maintaining the mechanistic structure of the PK/PD model.

All PK/PD analyses were conducted using Monolix (version 2024R1). Parameter estimation was carried out using a nonlinear mixed-effects modeling approach, employing the stochastic approximation expectation-maximization (SAEM) algorithm as implemented in the software. Simulations were performed using the Simulx module (MonolixSuite 2024R1), which enables model-based simulation based on the estimated PK/PD parameters.

### Pharmacokinetic model

**Fig 5:**
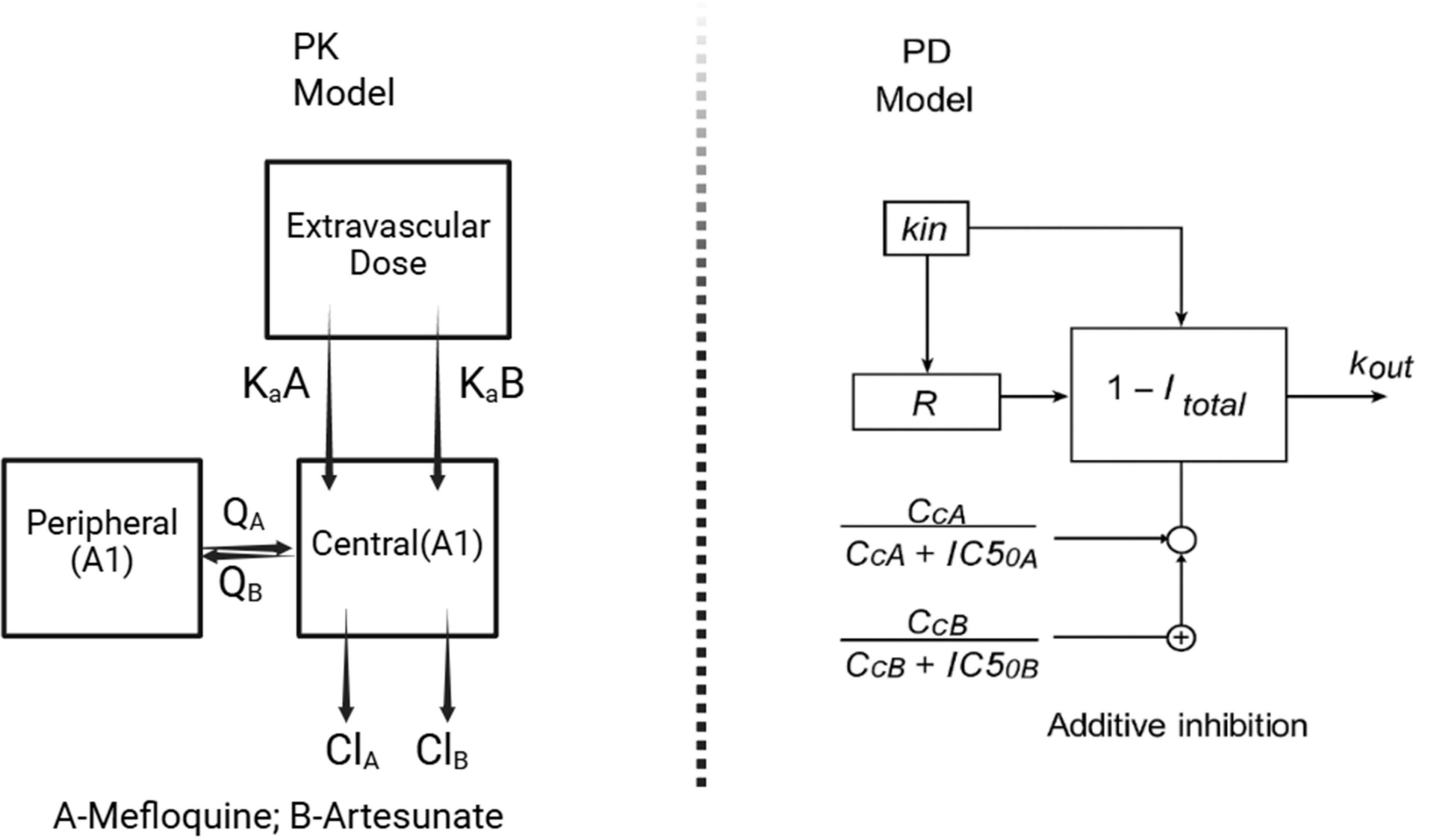
Schematic representation of the pharmacokinetic-pharmacodynamic (PK-PD) modeling framework used to characterize ASMQ combination therapy in mice. The pharmacokinetic component is described by a two-compartment model for each drug in the ASMQ regimen, capturing absorption, distribution, and elimination processes. The pharmacodynamic component employs a turnover model to describe parasite dynamics, linking drug exposure to parasite clearance and regrowth over time.

Pharmacokinetic data were obtained from 59 and 34 plasma samples for mefloquine and artesunate, respectively, and a stepwise modeling approach was employed accordingly. Various structural models were evaluated, including multicompartment disposition models with first-order absorption and either linear or nonlinear elimination, to identify the best-fitting model for each compound. For mefloquine, concentration data derived from the ASMQ combination therapy in mice were sufficient to support population pharmacokinetic modeling using a two-compartment disposition model with first-order absorption and linear elimination (Fig. 5). The estimated pharmacokinetic parameters were subsequently compared with those reported by McCarthy et al. *(*39) for model evaluation and were further supported by diagnostic goodness of fit plots, nonparametric bootstrap analysis. The pharmacokinetic data for artesunate were best described by a two-compartment model with first-order absorption and linear elimination. Direct external validation of the estimated parameters was not feasible due to the limited availability of preclinical pharmacokinetic data in mice. However, the estimated clearance and volume of distribution were allometrically scaled to human values to support the plausibility of the parameter estimates from Guidi et al. (9).

Model parameterization included clearance, intercompartmental clearance, and the volumes of distribution for both the central and peripheral compartments for mefloquine and artesunate. The absorption rate constants (Ka) were fixed at 0.31 h-¹ for mefloquine and 0.6 h-¹ for artesunate based on prior information (20). Different residual error models—proportional, additive, and combined—were evaluated to identify the best-fitting error structure.

### Pharmacodynamic model

The final pharmacokinetic model was integrated with a structural turnover pharmacodynamic model (Fig. 5), which characterizes the dynamic interplay between parasite production and elimination, with drug effects modeled as inhibition of parasite growth. Mechanism-based modeling approaches have previously been applied to describe the pharmacodynamic features of antimalarial drugs; however, our model extends this framework by simultaneously incorporating the effects of two agents: artesunate, a rapidly acting compound, and mefloquine, a slower-acting drug with sustained activity.

The turnover model effectively captured the combined parasite-killing effects of the ASMQ regimen and accounted for the temporal delay between drug administration and parasite clearance. Mefloquine was modeled to inhibit a hypothetical physiological intermediate via the turnover compartment, while artesunate was assumed to act on the same process described by Patel et al. (15), contributing an additive effect as represented in equation 1.


(1) $$\frac{dResponse}{dt}=Kin\left(1-\left(\frac{\left[MQ\right]}{IC50\_MQ+\left[MQ\right]}+\frac{\left[AS\right]}{IC50\_AS+\left[AS\right]}\;\right)\right)-Kout\times Response$$

Kin represents the zero-order rate constant for response (parasite) production. (Kin = R0*Kout)

R0 is the number of parasites present at the start of the treatment. IC₅₀ denotes the concentration of mefloquine or artesunate required to achieve 50% of the maximal inhibitory effect. Kout is the first-order rate constant describing parasite elimination due to drug-induced death.

Model performance was evaluated using diagnostic goodness-of-fit plots, including prediction versus observation and prediction-corrected visual predictive checks (pcVPC), along with information criteria, such as the Akaike Information Criterion (AIC) and Bayesian Information Criterion (BIC). Parameter precision was further assessed using nonparametric bootstrap analysis (*n* = 1,000 replicates), and relative standard errors (RSEs) were calculated as the ratio of the standard deviation of the bootstrap estimates to their mean values. Model selection was guided by lower AIC and BIC values, as well as acceptable parameter precision, defined as relative standard errors (RSEs) of less than 30% for fixed-effect parameters and less than or equal to 50% for random-effect parameters.

### Simulations to investigate dose regimen efficacy

The empirical PK/PD model parameters were used to simulate treatment scenarios for both artesunate monotherapy and ASMQ combination therapy. For the combination regimen, artesunate was administered at two dosing levels: 100 and 25 mg/kg, each in combination with 55 mg/kg of mefloquine. Additionally, artesunate monotherapy was simulated for a virtual population of 1,000 subjects receiving 100 mg/kg of artesunate.

## SUPPLEMENTAL MATERIAL

Supplemental material aac.01717-25-s0001.docx Tables S1 and S2.

ASM does not own the copyrights to Supplemental Material that may be linked to, or accessed through, an article. The authors have granted ASM a non-exclusive, world-wide license to publish the Supplemental Material files. Please contact the corresponding author directly for reuse.
