# Supplementary material for: Pharmacokinetic and pharmacodynamic modeling of anti-plasmodial drugs mefloquine plus artesunate: insights on translational application
Source: Antimicrob Agents Chemother. 2026 Feb 12;70(3):e01717-25. doi: 10.1128/aac.01717-25 (PMC12959152; doi:10.1128/aac.01717-25)
Supplement: Supplemental material — Tables S1 and S2. [file aac.01717-25-s0001.docx]

**Table S1.** Summary of efficacy of ASMQ in *P. berghei*-infected Swiss mice.

| **Groups** | **Dose (mg/kg)** | | **Efficacy** | | | **Cure** |
| --- | --- | --- | --- | --- | --- | --- |
|  | **AS** | **MQ** | | **% Inhibition^a^** | **Survival^b^** |  |
| Experiment #1 | **Follow-up of parasitemia reduction and animal survival** | | | | | |
| CTL | - | - | | - | 9 | 0/6 |
| Therapeutic (PK) | 100 | 55 | | 80.6 | 20^#^ | 2/6 |
| Adult | 41 | 20.5 | | 34.1 | 20^#^ | 0/6 |
| Pediatric | 2.5 | 5.0 | | N.D. | 8.5 | 0/6 |
| **Parasitemia in % (mean and S.D.) post-treatment** | | | | | | |
| Experiment #2 | 0 h | 8 h | | 24 h | 36 h | 48 h |
| CTL | 17.7±2.7 | 24.7±7.7 | | 32.9±11.7 | 27.6±6.9 | N.D. |
| AS (PK) | 20±7 | 15.6±2.8 | | 14.2±2.0 | 7.6±2.6* | 8.2±3.4* |
| Therapeutic (PK) | 17.4±3.1 | 15.3±12.4 | | 14.5±12.4 | 4.0±4.2* | 1.12±1.37*^,##^ |
| Experiment #3 | 0 h | 8 h | | 24 h | 36 h | 48 h |
| CTL | 11.8±1.98 | 11.2±3.7 | | 10.6±2.0 | 9.8±2.9 | 16.9±2.16* |
| Fixed ratio (FR) | 10.5±3.5 | 6.7±1.53 | | 6.6±0.95 | 1.42±0.71* | 1.17±0.63* |
| AS (FR) | 11.8±1.98 | 9.4±2.1 | | 7.9±1.14 | 4.7±1.67* | 6.5±3.1* |

^[a]^% Inhibition of parasitemia (mean and S.D.) in comparison to CTL. ^[b]^ Survival is the median in days. Abbreviations: CTL, untreated infected mice; DPI = days post-infection; FR = Fixed dose of 200 mg/kg MQ plus 100 mg/kg AS; AS (FR) = monotherapy using matched dose of FR of 100 mg/kg AS. Statistical analysis was conducted using the Mann-Whitney rank test. Unless indicated, ^##^ or **p*<0.05. ^#^*p*<0.05 (log-rank, Mantel-Cox test).

**Table S2.** Summary of dosing regimens for artesunate (AS) and mefloquine (MQ) administered to *Plasmodium berghei*–infected mice.

| **Drugs** | **Drug dose (mg/kg of animal weight)*** | |
| --- | --- | --- |
|  | **AS** | **MQ** |
| Therapeutic (PK) | 100 | 55 |
| Adult | 41 | 20.5 |
| Pediatric | 2.5 | 5.0 |
| Standard fixed ratio (FR) | 100 | 200 |
| AS (PK) | 100 | - |
| AS (FR) | 200 | - |

*Drug doses (mg/kg of animal weight) were evaluated under therapeutic pharmacokinetic (PK), adult, pediatric, and standard fixed-ratio (FR) treatment scenarios.
